# P05-08 Correlation between physical activity, Sleep componants and quality: In the context of type and intensity: A cross-sectional study among sudanese medical students

**DOI:** 10.1093/eurpub/ckac095.075

**Published:** 2022-08-29

**Authors:** Ahmed Abdelghyoum mahgoub, Shahenaz Satti Mustafa

**Affiliations:** Faculty of Medicine, Al-Neelain University, Khartoum, Sudan

**Keywords:** Work physical activity, Sleep Quality, Sleep Latency, lesiure time physical activity, Sedentary time

## Abstract

**Background:**

Physical activity during the day is composed of different domains, specifically work related, transportation, and recreation, physical activity. We aimed at studying the correlation between energy expenditure and the corresponding metabolic equivalent of task and sleep in the context of type of physical activity, general level of activity as to be low, moderate and vigorous and the intensity of activity either moderate or vigorous physical activity.

**Methodology:**

A cross-sectional study, participants were n = 273 enrolled from al-Neelain university faculty of medicine between January and April 2021 we used the global physical activity questionnaire to measure standard metabolic equivalent of task (MET) for participants for vigorous and moderate work MET, Transportation MET, Vigorous and moderate lesiure MET, and sedentary time. we used Pittsburgh sleep quality index to assess different components of sleep (subjective sleep quality, sleep latency, habitual sleep efficiency, sleep duration, sleep disturbances, use of medications, daytime dysfunction) and sleep quality.

**Results:**

Mean of Total-MET was (3533.36min/week) predominantly moderated work-MET (33%). Poor sleepers prevalence was high (62%). Moreover there was significant difference between good and poor sleepers in moderate work MET mean (876.36,1334.2 min/week) (p > 0.01). respectively. There was significant positive correlations between moderate work MET and roughly all sleep components rho = (0.196, 0.182, 0.132, 0.149)(p > 0.01, p > 0.01, p > 0.05, p > 0.05)respectively and sleep quality rho = (. 211)(p > 0.001). Vigorous-lesiure MET positively correlated with sleep latency rho = (0. 134)(p > 0.01). Total MET correlated with sleep latency, use of medications, and sleep quality in general. (0.134, 0.124, 0.133) (p > 0.05).

**Conclusion:**

Our results show that poor sleep quality is primarily influenced by the type and intensity of physical activity. Eliciting a dose-response effect of different domains, being deleterious for work related physical activity as work MET is of too low intensity or too long duration for maintaining or improving cardiorespiratory fitness and cardiovascular health subsequently imposing its deleterious effect. So in order to improve quality of life for university students, special strategies and policies that leverage ‘good sleep' quality are warranted by limiting work related physical activity and adding on well structured early morning exercises for University students thus improving cardiorespiratory fitness and subsequently sleep.

